# Editorial: Community series in new insights in sepsis pathogenesis and renal dysfunction: immune mechanisms and novel management strategies: volume II

**DOI:** 10.3389/fimmu.2023.1322571

**Published:** 2023-10-27

**Authors:** Patrick M. Honore, Alexandra Stasi, Vincenzo Cantaluppi

**Affiliations:** ^1^ Intensive Care Department, Université Catholique de Louvain (UCL) Louvain Medical School of Medicine, Centre Hospitalier Universitaire (CHU) UCL Mont-Godinne Namur, Yvoir, Belgium; ^2^ Nephrology, Dialysis and Transplantation Unit, Department of Precision and Regenerative Medicine and Ionian Area, University of Bari “Aldo Moro”, Bari, Italy; ^3^ Nephrology and Kidney Transplantation Unit, Department of Translational Medicine (DIMET), University of Piemonte Orientale (UPO), “Maggiore della Carità” University Hospital, Novara, Italy

**Keywords:** sepsis, COVID 19, subarachnoid bleeding, immunology, macrohemodynamics, autophagy, biomarkers, neutrophils and acupuncture

Sepsis, trauma, COVID-19-disease and subarachnoid hemorrhage (SAH) can cause severe organ failure, such as Acute Kidney Injury (AKI) and encephalopathy, among others (1). The mechanisms behind this induced organ failure are diverse and not limited to immune mechanisms, emphasized in Volume I of this series (2). In the second volume of the Research Topic “*New Insights in Sepsis Pathogenesis and Renal Dysfunction: Immune Mechanisms and Novel Management Strategies*” published in Frontiers in Immunology, we delve into a broader spectrum of mechanisms associated with life-threatening organ dysfunction. This includes exploring aspects such as macrohemodynamics and autophagy, which have been extensively studied in conditions such as severe trauma and sepsis (3). We also examine the intricate role of neutrophils and other contributing factors (2). Additionally, biomarkers for aiding in diagnosis and assessing organ function will be discussed. Furthermore, innovative approaches are considered, with a focus on acupuncture as a therapeutic intervention within this context, showing promise in mitigating organ damage and enhancing patient outcomes (4). Within this comprehensive exploration of mechanisms associated with life-threatening organ dysfunction in the second volume, noteworthy studies shed light on vital aspects.

One such study, led by Guo et al., has focused on AKI datasets GSE30718 and GSE44925. Their findings reveal that the hub gene Afamin (AFM) is significantly downregulated in AKI samples and correlates with the development of this syndrome. In another intriguing investigation, Zhao et al. explore the blood pressure target in sepsis-associated AKI (SA-AKI). Their conclusions suggest that, in order to ensure optimal renal perfusion, AKI patients with hypertension may benefit from a higher mean arterial pressure range (MAP), specifically in the range of 70-80 mmHg, as opposed to the traditional range of 65-73 mmHg.

Autophagy has gained attention for its crucial role in immune disorders after major trauma (3). Chen et al. demonstrated its significance in the early stages of trauma-induced immune disorders. Furthermore, their study showed a comprehensive single-cell immune profile for major trauma patients, unveiling how autophagy profoundly affects innate immune cell functions, providing insight into post-trauma immune dysregulation.

Sepsis-associated encephalopathy (SAE) has garnered significant research attention. A meta-analysis conducted by Hu et al. reveals a moderate association between elevated serum S100 calcium binding protein B (S100B) levels in septic patients and SAE, particularly concerning unfavorable outcomes including mortality. These findings suggest that serum S100B levels could serve as valuable diagnostic and prognostic biomarkers for SAE.

Recently, a compelling link has emerged between heightened levels of neutrophil extracellular traps (NETs) and adverse clinical outcomes in patients affected by COVID-19 disease. Kim et al. demonstrated that elevated NETs are closely tied to AKI, that in turn represents a robust predictor of mortality. This close connection between NETs and plasma von Willebrand factor raises the possibility of NETs playing a role in COVID-19-associated vasculopathy, potentially contributing to the development of AKI.

Pylephlebitis refers to an infective and suppurative thrombosis that affects the portal vein and its branches. When concurrent pylephlebitis and subarachnoid hemorrhage (SAH) occur in patients with sepsis, it presents a rare yet life-threatening situation. Managing both coagulation and bleeding simultaneously poses a significant challenge for clinicians. In a case report, Zhao et al. describe the successful treatment of an octogenarian with E. coli bacteremia who faced concurrent pylephlebitis, SAH, and multiple organ dysfunction syndrome (MODS). In such cases of critical complications, early and decisive use of LMWH (Low Molecular Weight Heparin) proves essential for resolving thrombosis and ultimately leads to a favorable prognosis.

Biomarkers are playing an increasingly vital role in the diagnosis of sepsis, as well as in understanding the intricate connections between genes and immune cells with differential expression in specimens from sepsis patients compared to healthy controls. In their study, Wang et al. pinpointed COMMD9, CSF3R, and NUB1 as potential genes that could serve as sepsis biomarkers—a hypothesis corroborated by ROC analysis. Furthermore, their research unveiled correlations between the expression of these three genes and the composition of immune cell infiltrates. Specifically, COMMD9 exhibited correlations with regulatory T cells, follicular helper T cells, CD8 T cells, and more. Similarly, CSF3R showed associations with regulatory T cells, follicular helper T cells, and CD8 T cells, while NUB1 correlated with regulatory T cells, gamma delta T cells, and follicular helper T cells. These collective findings not only identify promising new diagnostic markers for sepsis but also shed light on novel disease pathogenetic mechanisms, paving the way to potential therapeutic interventions.

In the realm of sepsis treatment, acupuncture has gained widespread acceptance and utilization, with notable advancements in understanding its mechanisms in recent years. In a breakthrough study, Yang et al. unveiled the pivotal role played by the cholinergic anti-inflammatory pathway of the vagus nervous system, the adrenal dopamine anti-inflammatory pathway, and the sympathetic nervous system in transmitting acupuncture’s therapeutic effects and suppressing systemic inflammation. Particularly in cases of Multiple Organ Dysfunction Syndrome (MODS), acupuncture serves as a protective shield against sepsis-induced organ damage. It achieves this by curbing excessive inflammatory responses, fortifying resistance against oxidative stress, safeguarding mitochondrial function, and diminishing apoptosis and tissue or organ damage.

The second volume of the “*New Insights in Sepsis Pathogenesis and Renal Dysfunction: Immune Mechanisms and Novel Management Strategies*” Research Topic in Frontiers in Immunology show the multifaceted landscape of sepsis and related conditions, encompassing mechanisms like immunology, macrohemodynamics, autophagy, and the impact of neutrophils. Studies by Guo et al., Zhao et al., Chen et al., Hu et al., Kim et al., Wang et al., and Yang et al. contribute valuable insights, revealing underlying mechanisms, potential diagnostic markers, novel pathways, and therapeutic interventions. This holistic perspective underscores the complexity of sepsis management, emphasizing the importance of diverse mechanisms and innovative strategies to improve patient outcomes.

## Author contributions

PH: Conceptualization, Writing – original draft, Writing – review & editing. AS: Writing – review & editing. VC: Writing – review & editing.

## References

[1] Kozlov AV Grillari J. Pathogenesis of multiple organ failure: the impact of systemic damage to plasma membranes. Front Med (Lausanne) (2022) 9:806462. doi: 10.3389/fmed.2022.806462 35372390 PMC8964500

[2] Stasi A Honore PM. Editorial: New insights in sepsis pathogenesis and renal dysfunction: Immune mechanisms and novel management strategies. Front Immunol (2023) 14:1176620. doi: 10.3389/fimmu.2023.1176620 36969189 PMC10031527

[3] Ren C Zhang H Wu TT Yao YM. Autophagy: A potential therapeutic target for reversing sepsis-induced immunosuppression. Front Immunol (2017) 8:1832. doi: 10.3389/fimmu.2017.01832 29326712 PMC5741675

[4] Matsuda A Jacob A Wu R Aziz M WL Y Matsutani T. Novel therapeutic targets for sepsis: regulation of exaggerated inflammatory responses. J Nippon Med Sch (2012) 79(1):4–18. doi: 10.1272/jnms.79.4 22398786

